# A Novel Autoantibody Induced by Bacterial Biofilm Conserved Components Aggravates Lupus Nephritis

**DOI:** 10.3389/fimmu.2021.656090

**Published:** 2021-03-25

**Authors:** Wenyan Fu, Yu Liu, Fangjie Liu, Chenghua Liu, Jingjing Li, Jiali Niu, Peng Han, Dan Xu, Jiaojiao Hou, Yuanfang Ma, Jiannan Feng, Zhanguo Li, Rong Mu, Guang Yang

**Affiliations:** ^1^ Beijing Institute of Pharmacology and Toxicology, Beijing, China; ^2^ State Key Laboratory of Toxicology and Medical Countermeasures, Beijing, China; ^3^ Joint National Laboratory for Antibody Drug Engineering, Henan University, Kaifeng, China; ^4^ Department of Rheumatology and Immunology, People’s Hospital, Peking University, Beijing, China

**Keywords:** bacterial biofilm, DNABII, autoantibody, lupus nephritis, protein disulfide isomerase

## Abstract

Systemic lupus erythematosus (SLE) is a systemic autoimmune disease with multiple autoantibody production and often affects the kidneys, known as lupus nephritis. However, the mechanism underlying lupus nephritis development is unclear. Biofilms that protect bacteria from stress are ubiquitous in almost every environment. Here, we identified that a conserved peptide (HU1) derived from DNABII proteins, one of major bacterial biofilm components, was specifically recognized by sera from about 47% patients with SLE. Moreover, the serum anti-HU1 levels showed a significant positive correlation with lupus nephritis occurrence. Presence of antibodies against HU1 in pristane-induced mice aggravated lupus nephritis, although these antibodies also attenuated bacterial biofilm formation. We further identified that antibodies against HU1 cross-recognized protein disulfide isomerase (P4HB) located on the renal cell surface and inhibited the activities of this enzyme. Our findings reveal a novel mechanism underlying the development of lupus nephritis triggered by bacterial biofilms.

## Introduction

Systemic lupus erythematosus (SLE) is a systemic autoimmune disease characterized by the presence of numerous autoantibodies and immune complex formation (1). Well-known autoantibodies including antinuclear antibodies (ANA) and anti-double stranded DNA (anti-dsDNA) have been used for the diagnosis of SLE. As a chronic inflammatory disease, SLE can affect any organ, but most often injures the kidney. Lupus nephritis (LN) is characterized by renal deposition of immune complexes and considered as a major risk factor for morbidity and mortality in SLE (2). Although deposition of autoantibody and immune complexes in renal compartments contributes to the development of LN, the pathogenesis of LN is far from clear (3).

Both genetic and environmental triggers contribute to SLE (4). Bacterial infection is an important environmental trigger for lupus onset as well as the leading cause of morbidity and mortality in patients with SLE. About 20–55% of SLE patients’ deaths are attributable to infectious diseases (5). Bloodstream infections, urinary tract infections, pneumonia and soft tissue infections are often observed in patients with SLE (6). Several bacterial pathogens such as *Staphylococcus aureus*, *Streptococcus pneumoniae*, *Escherichia coli*, or *Pseudomonas aeruginosa* are frequently isolated from patients with SLE (7, 8).

More than 80% of all bacterial infections include a necessary phase within the disease course, called biofilm phase (9). Bacterial biofilms are known to form on mucosal surfaces of the human body (10). The structure of bacteria biofilms is complex, with bacterial communities surrounded by the extracellular matrix (ECM), which protects bacteria from antibiotics and host immune defenses (11). ECM is composed of extracellular polysaccharide, extracellular protein, and extracellular DNA (eDNA) (12). Among these components, eDNA is essential for structural integrity and stability to the biofilm *via* interaction with DNA binding proteins (13, 14).

The major eDNA binding proteins in bacterial biofilms are members of the DNABII family which are ubiquitous among eubacteria (15). DNABII proteins include histone like protein (HU) and integration host factor (IHF). HU is a hetero- or homodimer of each subunit and IHF functions through a heterodimer of IHFA and IHFB (16). The subunits of HU and IHF have a highly conserved sequence homology (15).

As human immune system is considered to be exposed to biofilm components throughout life and as DNA interaction with protein antigens is reported to induce autoimmune disorders (6, 17), we investigated whether DNABII proteins are involved in the induction of autoimmunity. In the present study, we had demonstrated that a conserved peptide derived from DNABII proteins specifically recognized by sera from patients with SLE, especially in those with lupus nephritis (LN). Our following study showed that antibodies against this peptide cross recognized a self-antigen, which was involved in the development of LN.

## Materials and Methods

### Patients, Ethics Approval and Consent to Participate

Patients diagnosed in the Department of Rheumatology and Immunology Peking University People’s Hospital from 2008 to 2015 were randomly selected. All patients were diagnosed by the established disease criteria sets (18–20).

Whole blood samples were taken from all participants after written informed consent was obtained. The study was approved by the ethics committee of the Peking University People’s Hospital (2019PHB156-01). All animal experimental protocols of the study are in accordance with the national guidelines for the use of animals in scientific research “Regulations for the Administration of Affairs Concerning Experimental Animals” and were approved by the Animal Care and Use Committee of Beijing Institute of Pharmacology and Toxicology.

### The Generation of HU1 and HU2 Peptide

HU1 and HU2 peptides (including BSA and KLH conjugated peptides) were chemically synthesized by Anhui Guoping Pharmaceutical Co., Ltd., China.

### Detection of Specific Antibody in Human Serum

Enzyme linked immunosorbent assay (ELISA) was performed to detect the specific antibodies in human serum. Peptide or proteins were coated and incubated with the diluted serum (1:50) from patients with autoimmune disease and healthy individuals at 37°C for 1 h, and HRP-conjugated goat-anti-human IgG (1:40,000, Jackson Immuno, 109-035-003) was added to each well as secondary antibody. The levels of specific antibodies were determined by measuring the absorbance at 450 nm.

### Preparation of Rabbit Polyclonal Antibody

For preparation of rabbit anti-HU1 antibody, Freund’s adjuvant was used for enhancing immune response. Rabbits were individually injected subcutaneously on the back or hind legs with 0.2 mg different antigens (KLH-conjugated HU1, SaHU, PaIHFB and recombinant human P4HB) emulsified in Freund’s complete adjuvant on day 1, and four times of boost immunization were performed on days 7, 14, 21 and 28 with mixture of antigens and Freund’s incomplete adjuvant. Four days after last immunization, all serum were taken. Purification of total IgG using Protein A-resin was required first, and specific IgG were individually purified using the affinity resins which were prepared by conjugating antigens to SulfoLink Coupling Resin (Pierce) according to the manufacturer’s instructions.

### Expression of DNABII Protein and Mutant Protein

The genomic DNA of *S. aureus* 8325 and *P. aeruginosa* PAO1 were extracted individually and PCR primers were designed according to the gene sequences of Sa.HU (Gene ID: 3920245), Pa.IHFA(Gene ID: 57390173) and Pa.IHFB(Gene ID:882561). The PCR products were cloned into the NheI–XhoI-digested pET-28a plasmid. The recombinant expression plasmids were transformed into *E. coli* BL21 (DE3) cells. LB medium containing 0.2 mg/ml Kanamycin was used for selection and overexpression was then induced by adding 0.5 mM isopropyl β-D-1-thiogalactopyranoside (IPTG). After additional incubation for 8 h at 37°C, the harvested cells were resuspended in lysis buffer (20 mM Imidazole, 20 mM Na_3_PO_4_, 0.5 M NaCl, pH 7.4) and lysed by ultrasonication. The cell lysate was loaded onto a Ni^2+^–NTA resins (TransGen Biotech, DP101-01) and washed with lysis buffer containing 50 mM imidazole. The resin-bound protein was eluted using 500 mM imidazole. Finally, the buffer containing single protein was changed to PBS by dialysis. The purity of DANBII protein was estimated by SDS-PAGE.

The mutant protein Sa.HUm, Pa.IHFAm, and Pa.IHFBm were constructed by replacing the HU1 region in the proteins with six alanine by PCR. The cloning and expression of mutant protein were performed by the methods as described above.

### Biofilm Formation Assay and Quantification

Biofilm formation was determined by the ability of cells to adhere to the base of 96-well (flat-bottom) polystyrene microtiter dishes (NEST Biotechnology). The overnight culture of *S. aureus* 8325 was diluted to 10^6^ CFU/ml in TSB medium. The diluted *S. aureus* 8325 (190 μl) and Rb-anti-HU1, control rabbit IgG or sterilized PBS(10 μl respectively) were added to each well. Plates were incubated at 37°C for 4 h. About 100 μl 1% solution of crystal violet (CV) was added to each well. The plates were then incubated at room temperature for approximately 15 min. After three times of washing with distilled water, CV-stained bacterial biofilm was solubilized with 30% acetic acid (100 μl/well). Biofilm formation was determined by measuring the absorbance at 595 nm.

For confocal microscopy detecting, *S. aureus* 8325 strain carrying green fluorescence protein (*S. aureus*-GFP) was used. The overnight culture of *S. aureus*-GFP was diluted to 10^6^ CFU/ml in TSB medium. The diluted *S. aureus*-GFP (190 μl) and Rb-anti-HU1, control rabbit IgG or sterilized PBS (10 μl respectively) were added to each well. Plates were incubated at 37°C for 6 h. After three times of washing with sterilized PBS, the bacteria biofilm was analyzed using PE UltraView VoX Turntable Confocal Microscope according to manufacturer instructions.

### Mice and Analysis of Immunized Mice

Six- eight weeks old wild-type female BALB/c mice were obtained from Beijing Vital River Laboratory Animal Technology Co., Ltd. Before induced by pristane i.p (500 μl/mouse), mice (10 mice in each group) were immunized with KLH-HU1 or KLH every 3 weeks for a total of six times. For each mouse, urine and serum samples were collected every two weeks. Urine total protein was determined by Easy Protein Quantitative Kit (TransGen Biotech). The levels of urinary KIM-1 (kidney injury molecule-1) were measured by Mouse KIM-1 ELISA Kit (ab213477, Abcam). The anti-dsDNA antibodies in the serum were quantified with anti-dsDNA IgG ELISA kit (EUROIMMUN). All mice were sacrificed at week 30 after pristane injection, and the kidney were obtained to take pathological examination by HE staining and immune complex detection by fluorescence microscopy.

### Histological Analyses

The method to score kidney pathology was as described before (21, 22). Kidneys from different group of mice were fixed in 4% paraformaldehyde for overnight and then embedded in paraffin. Paraffin sections (5 μm) were stained with HE. Pathological changes in the kidney were assessed by evaluating glomerular activity (i.e., glomerular proliferation, karyorrhexis/fibrinoid necrosis, cellular crescents, hyaline deposits, and inflammatory cells) and tubulointerstitial activity (i.e., interstitial inflammation, tubular cell necrosis, and flattening and tubular distension). Sections were scored using a 0–3 scale for glomerular activity, as follows: 0 = no lesions, 1 = lesions in>25% of glomeruli, 2 = lesions in 25–50% of glomeruli, and 3 = lesions in >50% of glomeruli. Tubulointerstitial activity was scored using a 0–4 scale, as follows: 0 = no lesions, 1 = lesions in 1–10%, 2 = 11–25%, 3 = >25-50% and 4 = >50–100%. The scores for individual pathological features were summed

For immunofluorescence studies, paraffin kidney sections were blocked in a solution of 1% BSA in PBS at room temperature for 1 h and stained with goat anti-mouse IgG antibody labeled with Alexa Fluor^®^ 488 (1:100; Biolegend, 405319) for 1 h. Images were acquired using PE UltraView VoX Turntable Confocal Microscope according to manufacturer instructions.

### Immunofluorescence Confocal Microscopy

To determine the recognition of Rb-anti-HU1 to the membrane protein of HEK293T cells and mouse primary kidney cells, which were incubated with Rb-anti-HU1 IgG or rabbit control IgG at a concentration of 10 μg/ml. The DyLight^™^ 488 conjugated donkey anti-rabbit IgG (Biolegend) antibody was used as the detecting antibody. After washing three times with PBS, cells were coated on glass chamber slides. The nuclei were stained by DAPI. The labeled cells were analyzed using PE UltraView VoX Turntable Confocal Microscope.

### Specific Binding of Anti-HU1 and P4HB

ELISA was performed to determine the binding of anti-HU1 and human/mouse P4HB individually. Human or mouse P4HB was coated individually (0.5 μg/well) and incubated with different concentration Rb-anti-HU1 antibody (concentration varied from 10 to 0.625 μg/ml) at 37°C for 1h. HRP-conjugated goat-anti-rabbit antibodies (1:40,000, Jackson Immuno,111-036-144) were added to each well as secondary antibody and incubated at room temperature for 0.5 h. The level of antibodies bond with the coated protein was determined by measuring the absorbance at 450 nm.

Competitive ELISA was performed to detect the specific binding of Rb-anti-HU1 to human P4HB. Human P4HB (0.5 μg/well) was coated at 4°C for overnight. Different concentrations of HU1 peptides (0, 0.12, 1.2, 12, and 120 μg/ml) were pre-incubated with Rb-anti-HU1 antibody (final concentration of 2.5 μg/ml) at 37°C for 0.5 h. The pre-incubated mixture was added to the coated wells (100 μl/well) and incubated at 37°C for 1 h. HRP-conjugated goat-anti-rabbit antibody (1:40,000, Jackson Immuno,111-036-144) was added to each well as secondary antibody and incubated at room temperature for 0.5 h. The level of antibodies bond with the coated protein were determined by measuring the absorbance at 450 nm.

### Measurement of Reductase P4HB Activity

P4HB activity was assayed by measuring the aggregation of reduced insulin B chain at 630 nm in the presence of DTT (23). Briefly, recombinant human P4HB protein was incubated with anti-HU1 antibody at 37°C for 1 h in sodium phosphate buffer (100 mM sodium phosphate, 2 mM EDTA, 8 μM DTT, pH 7.0). After the incubation, the P4HB protein mixture was added to the reaction mixture consisting of DTT (500 μM) and bovine insulin (130 μM). The reduction reaction was catalyzed by P4HB at room temperature, and the resulting aggregation of reduction insulin B chains was measured at 630 nm (24).

### Statistical Analysis

Statistical analyses were performed with two-tailed unpair Student’s t test, correlation analysis and two-way ANOVA test using GraphPad Prism 7 software. P >0.05 was considered nonsignificant. All data are presented as means ± SD.

## Results

### A Peptide Derived From the Conserved Domain of DNABII Proteins Is Specifically Recognized by Sera From Patients With SLE

As bacterial biofilms *in vivo* are directly exposed to the human immune system and DNABII proteins are the major DNA binding proteins in biofilms, we examined whether these conserved proteins could trigger immune responses. Three recombinant DNABII proteins (HU protein from *S. aureus*—Sa.HU and IHFA/B from *P. aeruginosa*—Pa.IHFA/B) were expressed in *E. coli* respectively (**Figure S1A**). We then determined the presence of antibodies against these DNABII proteins in patients with rheumatoid arthritis (RA), SLE and primary Systemic sclerosis (pSS) and in healthy donors by ELISA. Sera from most patients with these autoimmune diseases as well as healthy donors were found to recognize the three DNABII proteins respectively, which suggested the bacterial biofilms were widely present in the host. The level of anti-HU and anti-IHFB in serum from patients with SLE were higher than that in healthy donors. However, there was no difference observed among patients with three autoimmune diseases (**Figure S1B**).

**Figure 1:**
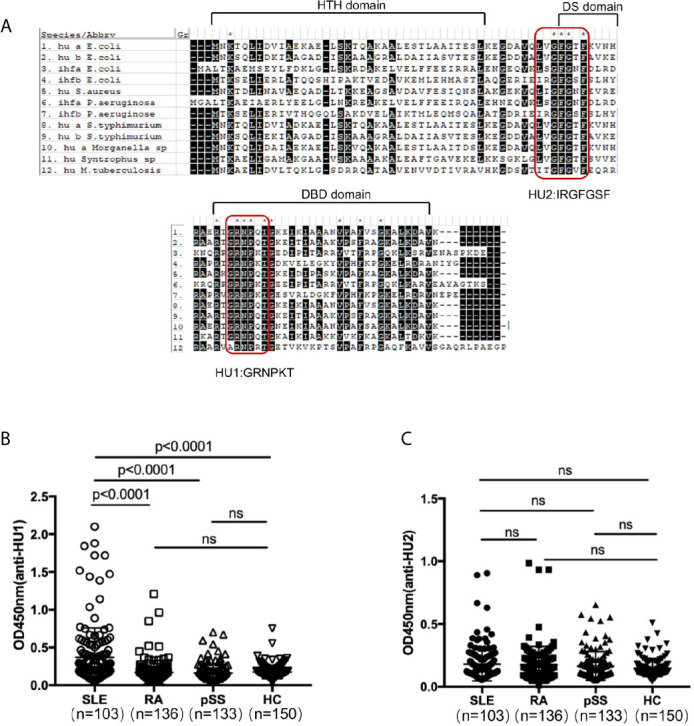
A peptide derived from the conserved domain of DNABII proteins was specifically recognized by sera from patients with SLE. **(A)** Sequence homology alignment of DNABII proteins from different bacteria. The domain of the secondary structure is labeled above the alignment: helix–turn–helix (HTH) domain, the dimerization signal (DS) and the DNA-binding domain (DBD). Highly conserved residues are highlighted in black. Two highly conserved domains were designed and named HU1 (GRNPKT), HU2 (IRGFGSF). Sequences were obtained from Uniprot (https://www.uniprot.org) and alignments were set up with MEGA 5.1 software. Levels of anti-HU1 **(B)** and anti-HU2 **(C)** in sera from patients with autoimmune diseases and from healthy controls. Each point represents a measurement for an individual (Systemic lupus erythematosus, SLE, n = 103; Rheumatoid Arthritis, RA, n = 136; primary Systemic sclerosis, pSS, n = 133; healthy donor control, HC, n = 150). Statistical analyses were performed with two-tailed unpair Student’s t test using GraphPad Prism 7 software. P > 0.05 was considered nonsignificant.

DNABII proteins have high sequence homology and conserved domains. We further detected the recognition of the different domains of these proteins by individual sera. Firstly, we designed two peptides from their highly conserved domains and named them as HU1(GRNPKT) and HU2 (IRGFGSF) by sequence homology analysis (**Figure 1A**). In our investigation, it was found that HU1 was specifically recognized by sera from patients with SLE, but not HU2. The level of anti-HU1 in sera from patients with SLE was significantly higher than that in patients with other autoimmune diseases and healthy controls (**Figures 1B, C** **)**.

### HU1 Is a Protective Epitope of DNABII Proteins

**Figure 2:**
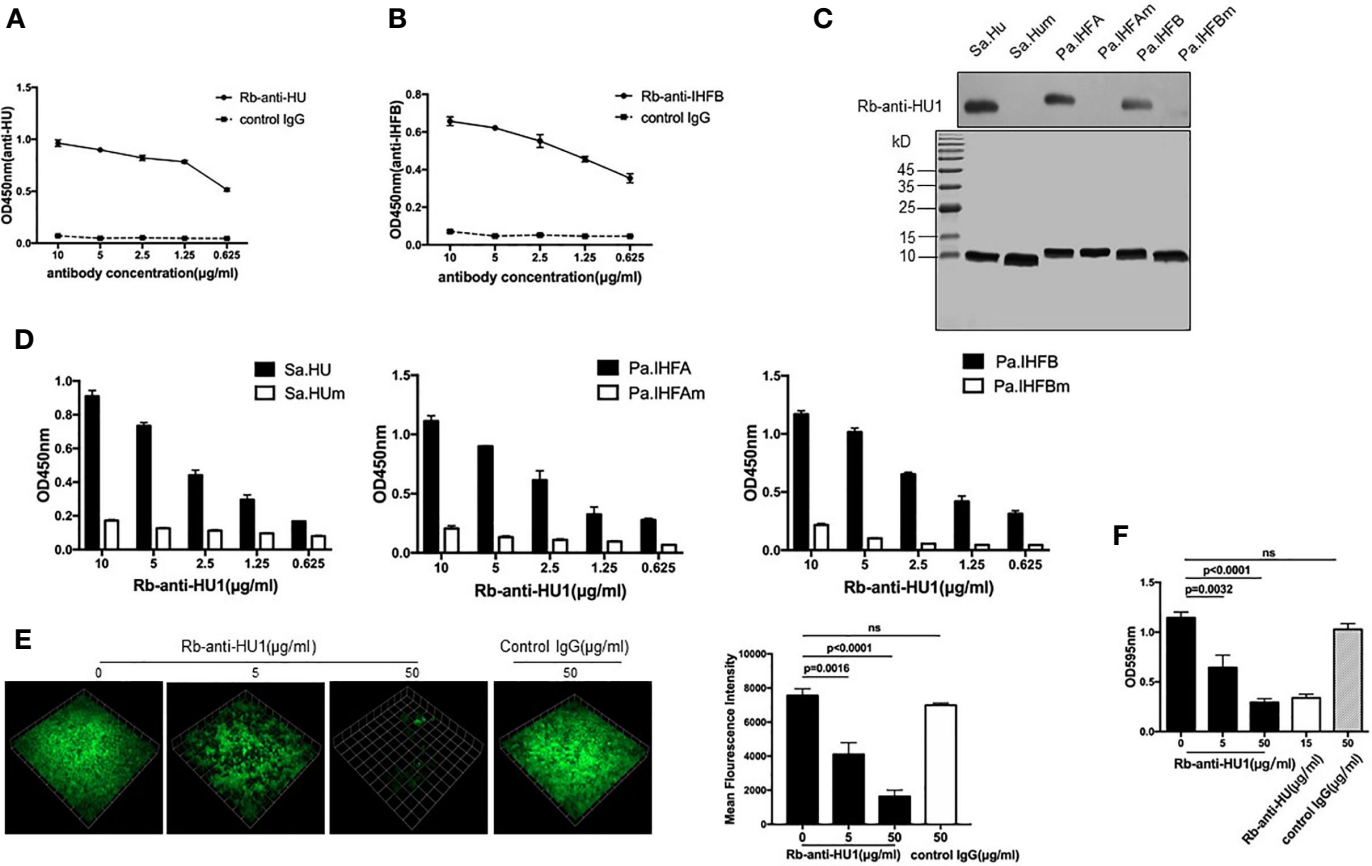
HU1 is a protective epitope of DNABII proteins. Interaction between HU1 peptide and Rb-anti-HU **(A)** and Rb-anti-IHFB **(B)** was determined by ELISA. **(C)** Western blot assay (upper) demonstrated the binding of Rb-anti-HU1 with DNABII proteins but not with mutant proteins. SDS-PAGE (lower) showed that the same quality proteins were used in the analysis. **(D)** ELISA detecting the recognition of Rb-anti-HU1 with DNABII proteins and mutants. **(E)** 3D confocal laser scanning microscopy images of GFP expressing S. aureus (green) biofilms with different concentrations of Rb-anti-HU1 treatment (left). One unit presents 33.47 μm. The mean fluorescence intensity was analyzed using Volocity Demo software (right). Images are representative of three independent experiments. **(F)** Crystal violet assay of *S. aureus* biofilms grown in 96-well plates with different concentrations of Rb-anti-HU1. Data are representative of three independent experiments and are shown as mean ± SD. Statistical analyses were performed with two-tailed unpair Student’s t test using GraphPad Prism 7 software. P >0.05 was considered nonsignificant.

Considering that HU1 can interact with antibodies from patients with SLE, we assumed that this peptide is one of the epitopes of DNABII proteins. We first prepared rabbit polyclonal antibodies against HU1 (Rb-anti-HU1) by vaccination using HU1 conjugated with Keyhole limpet hemocyanin (KLH-HU1) (**Figure S2A**). Rabbit polyclonal antibodies against DNABII proteins (Sa.HU and Pa.IHFB) (Rb-anti-HU and Rb-anti-IHFB) were also prepared, respectively (**Figure S2B**). The results of binding ELISA showed that both Rb-anti-HU and Rb-anti-IHFB recognized the HU1 peptide (**Figures 2A, B** **)**.

To further confirm that HU1 is an epitope of DNABII proteins, we expressed three mutant DNABII proteins (Sa.HUm, Pa.IHFAm, and Pa.IHFBm) with the region corresponding to HU1 deleted. The interaction between Rb-anti-HU1 and the wild-type and mutant DNABII proteins was then determined by western blot and ELISA. Rb-anti-HU1 was found to recognize wild-type DNABII proteins but not the mutant proteins (**Figures 2C, D** **)**.

As an epitope of DNABII proteins, HU1 peptide triggered the production of specific antibodies. Antibodies targeting DNABII proteins are reported to inhibit the formation of bacterial biofilms (16), we therefore determined the effect of Rb-anti-HU1 on *S. aureus* biofilm. Presence of Rb-anti-HU1 was found to significantly decrease the formation of the *S. aureus* biofilm (**Figures 2E, F** **)**. These results suggested that HU1 is one of the protective epitopes of DNABII proteins.

### Antibodies Against HU1 Aggravate Lupus Nephritis in the Pristane-Induced SLE Murine Model

**Figure 3:**
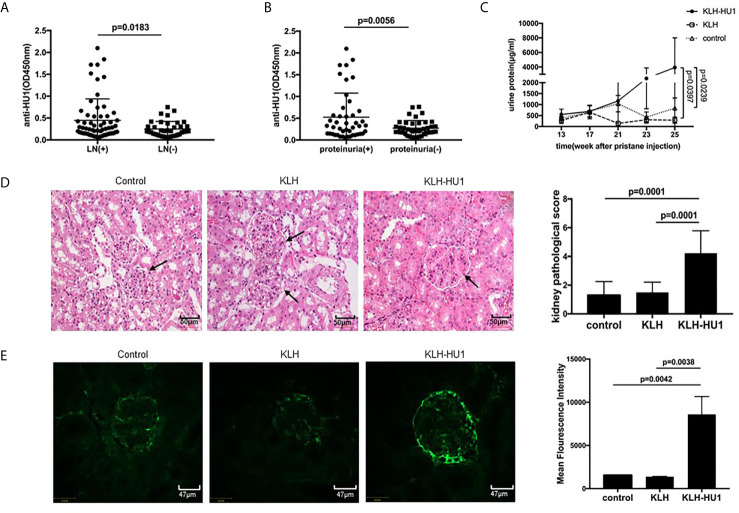
Antibodies against to HU1 aggravated lupus nephritis in a pristane-induced SLE murine model. **(A)** Correlation between anti-HU1 antibody levels and occurrence of LN in patients with SLE (LN+, n = 63; LN−, n = 38). **(B)** Correlation between anti-HU1 antibody levels and 24-hour urine protein (g/day) in patients with SLE. A level of 24-hour urine protein (g/day) higher than 0.3 g/day was considered positive(proteinuria+, n=45; proteinuria−, n = 42). **(C)** The urine protein content of KLH-HU1 immunized mice and control groups mice was determined at the indicated time (n = 10 for each group). **(D)** Kidney histopathology was analyzed at week 30 post-pristane injection (control, n = 10; KLH, n = 10; KLH-HU1, n = 9). **(E)** Immune complex deposition in glomeruli was determined using Alexa Fluor^®^ 488 labeled anti-mouse IgG antibody at week 30 post-pristane injection (control, n = 10; KLH, n = 10; KLH-HU1, n = 9). Statistical analyses were performed with two-tailed unpair Student’s t test using GraphPad Prism 7 software. P >0.05 was considered nonsignificant. All data are shown as mean ± SD.

As antibodies against HU1 are specifically present in patients with SLE, we investigated the pathological significance of their presence. We first analyzed the correlation between the level of anti-HU1 in sera from patients with SLE and their clinical features. The level of anti-HU1 antibodies in the sera of SLE patients with LN was significantly higher than that in patients without LN (**Figure 3A**). The same result was also reflected in the 24-hour urine protein (g/day), which indicates kidney damage and impaired renal function (**Figure 3B**). To confirm that LN was correlative with anti-HU1 but not with other cross-reactive antibody, we further analyzed the level of anti-HU2 antibodies in SLE patients with LN and patients with SLE alone, or SLE patients with proteinuria and without proteinuria. It was found that there was no observed difference between these groups (**Figures S3A**, **S3B**). Moreover, the level of anti-HU1 antibody was positively correlated with the units of anti-dsDNA antibody in the sera from patients with SLE (**Figure S3C**). No correlation with other clinical features was observed (**Figures S3D, E**).

Kidney is the most commonly affected organ in SLE and LN is a major risk factor for overall morbidity and mortality (2, 25). We further investigated whether anti-HU1 is involvement in the pathogenesis of LN. Wild-type female BALB/C mice were immunized with KLH-HU1 to produce specific antibodies, and we found only mice immunized with KLH-HU-1 produced the antibodies against HU-1 (**Figure S3F**); the mice were then injected intraperitoneally with pristane to construct a lupus-like murine model. The level of urine protein was determined at different time points after pristane injection. We found that the level of urine protein in KLH-HU1 immunized mice was rapidly increased compared with that in control groups. The level of urine protein in the KLH-HU1 group was significantly higher than that in the control groups at the 23rd and 25th weeks after pristane injection (**Figure 3C**). As expected, hematoxylin and eosin staining showed that the kidney of KLH-HU1 immunized mice had a thickened renal basement membrane, and poor opening of the vascular loop. Pathological changes in the kidney were assessed according to the methods described by Bignon A et al. (21, 22). Histological analysis revealed effects of anti-HU1 on tubulointerstitial and glomerular injuries (**Figure 3D**). Furthermore, increased IgG deposition was found in the kidneys from KLH-HU1 mice compared with that in the control groups (**Figure 3E**). However, no difference was detected in the anti-dsDNA antibody levels among the mice from the three groups (**Figure S3G**).

### P4HB, a Protein Disulfide Isomerase (PDI), as a Target Autoantigen

**Figure 4:**
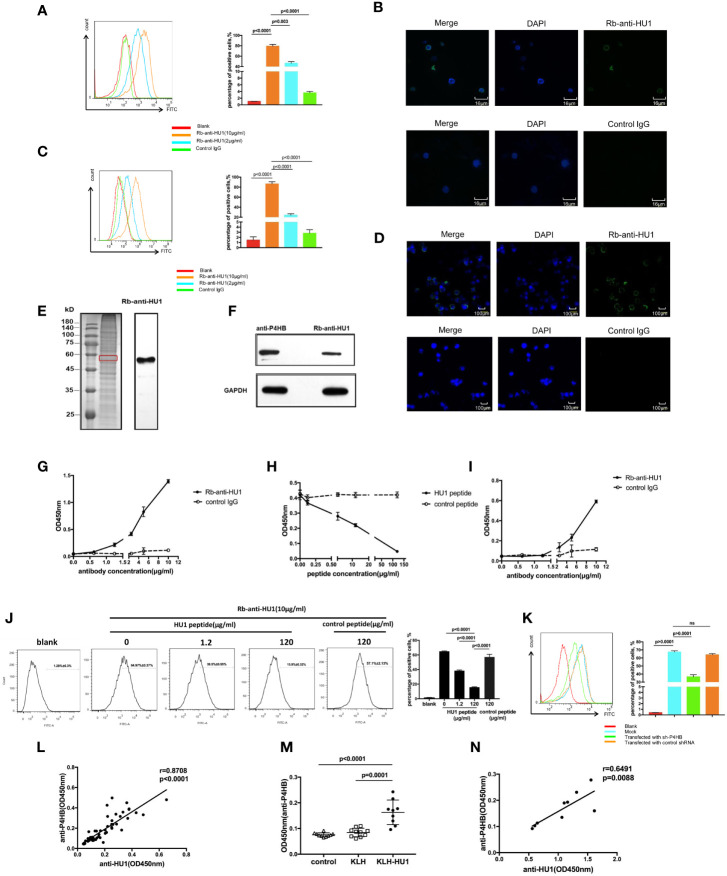
The protein disulfide isomerase, P4HB, as a target autoantigen. **(A)** Binding of Rb-anti-HU1 to mouse primary kidney cells was measured by flow cytometry. Live mouse primary kidney cells were stained with Rb-anti-HU1 (0 μg/ml-red, 2 μg/ml-blue, 10 μg/ml-orange), and then detected with DyLight™ 488 labeled donkey anti-rabbit IgG. Flow cytometry analysis was performed on FACSCalibur (Becton Dickinson). Data were processed using FlowJo software. Data are representative of three independent experiments and are shown as mean ± SD. **(B)** Confocal microscopy showed that Rb-anti-HU1 (10 μg/ml) recognizes a member antigen on mouse primary kidney cells. **(C)** Binding of Rb-anti-HU1 to HEK293T cells was measured by flow cytometry. HEK293T cells were stained with Rb-anti-HU1 (0 μg/ml-red, 2 μg/ml-blue, 10 μg/ml-orange), and then detected with DyLight™ 488 labeled donkey anti-rabbit IgG. Flow cytometry analysis was performed on. FACSCalibur (Becton Dickinson) and data were processed using FlowJo software. Data are representative of three independent experiments and are shown as mean ± SD. **(D)** Confocal microscopy showed that Rb-anti-HU1 (10 μg/ml) recognizes a member antigen on HEK293T cells. **(E)** HEK293T cells total proteins were extracted and detected by western blot using Rb-anti-HU1 antibody. The band labeled in the red square indicates the specific band recognized by Rb-anti-HU1. Data represent one of three independent experiments. **(F)** Bands recognized by Rb-anti-HU1 and Mo-anti-P4HB were detected from total proteins of HEK293T cells by western blot. The interaction between Rb-anti-HU1 and human P4HB **(G)** and mouse P4HB **(I)** was measured by ELISA. Data are shown as the mean ± SD. **(H)** Specific binding of Rb-anti-HU1 to human P4HB was blocked by HU1 peptide in a concentration-dependent manner. Data are shown as mean ± SD. **(J)** Specific binding of Rb-anti-HU1 to native P4HB on mouse primary kidney cell surface was blocked by HU1 peptide in a concentration-dependent manner. Data are representative of three independent experiments and are shown as mean ± SD. **(K)** P4HB expression was knocked down by a specific small hairpin RNA(sh-P4HB). Flow cytometry was then performed to detected the specific binding of Rb-anti-HU1 (5 μg/ml) to P4HB on the surface of HEK293T cells by flow cytometry. Data are shown as mean ± SD. **(L)** Correlation between the anti-HU1 antibody and anti-P4HB antibody in sera from patients with SLE. Each point represents a measurement for an individual patient (n = 62). **(M)** Detection of anti-P4HB titer in the sera of mice immunized with KLH-HU1 and control groups by ELISA at week 23 post-pristane induction (control, n = 10; KLH, n = 10; KLH-HU1, n = 9). **(N)** Correlation between anti-HU1 antibody and anti-P4HB antibody in sera from mice immunized with KLH-HU1 (n = 9). Data are presented as means ± SD. The differences between two groups were statistically analyzed with two-tailed unpair Student’s t test using GraphPad Prism 7 software. The correlation between two indicators were statistically analyzed with correlation analysis using GraphPad Prism 7 software. P >0.05 was considered nonsignificant.

Antibodies against pathogen proteins that cross-react with self-antigens become autoantibodies. To further investigate the role of anti-HU1 in the pathogenesis of LN, we performed flow cytometry to examine whether anti-HU1 bond to mouse primary kidney cells. Rb-anti-HU1 was found to specifically bind mouse primary kidney cells, suggesting that self-antigens on the cell surface were recognized by Rb-anti-HU1 (**Figure 4A**). Cell surface staining by Rb-anti-HU1 was also confirmed by confocal microscopy (**Figure 4B**). Upon further investigation, we found that Rb-anti-HU1 could also recognize self-antigens on the surface of HEK 293T, a human embryonic kidney cell line (**Figures 4C, D** **)**. We then investigated the corresponding human antigens cross-recognized by anti-HU1. The total proteins of HEK 293T cells were extracted and stained with anti-HU1, and demonstrated a specific band with an apparent molecular weight of ~58 kDa (**Figure 4E**). The corresponding band in SDS-PAGE was identified by mass spectrometry. There were totally 193 potential proteins identified with different score. In top 20 candidates according to score (**Table S3**), P4HB was the only transmembrane protein with ~58 kDa molecular weight. P4HB is a multifunctional protein disulfide isomerase that catalyzes oxidation, reduction, and heterogeneity of functional protein disulfide bonds, and thereby is involved in a variety of physiological and pathological processes.

In further investigation, the specific bands recognized by Rb-anti-HU1 and anti-P4HB were found to have the same molecular weight (**Figure 4F**). We performed ELISA to analyze the interaction between Rb-anti-HU1 and human P4HB protein (**Figure 4G**). Rb-anti-HU1 specifically recognized human P4HB, and this interaction was inhibited by HU1 peptide in a concentration-dependent manner (**Figure 4H**). Specific recognition of mouse P4HB protein by anti-HU1 was also observed (**Figure 4I**). We further detected whether the interaction between anti-HU1 and native P4HB was specifically inhibited by HU1 peptide by flow cytometry. A non-related hexapeptide (not HU2) was used as the negative control. It was found that the interaction between anti-HU1 and P4HB located on mouse primary kidney cell membrane was specifically blocked by HU1 but not by the control peptide (**Figure 4J**). Furthermore, we found that the level of Rb-anti-HU1 binding to the cell membrane decreased after P4HB expression in 293T cells was knocked down with a specific small hairpin RNA (**Figures 4K**, **S4**). The positive correlation between anti-HU1 and anti-P4HB antibody levels in the serum of SLE patients further demonstrated cross reaction of anti-HU1 with P4HB (**Figure 4L**). As expected, mice immunized with KLH-HU1 produced specific antibodies against mouse P4HB that were positively correlated with the level of anti-HU1 (**Figures 4M, N** **)**.

**Figure 5:**
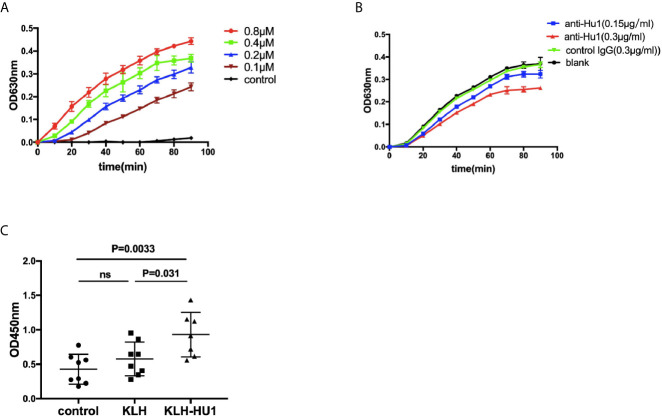
The inhibitory effect of anti-HU1 on the enzymic activities of P4HB. **(A)** Insulin aggregation assay was performed to determine the activity of recombinant human P4HB. **(B)** Rb-anti-HU1 (0.15 μg/ml-blue, 0.3 μg/ml-red) inhibited the activity of recombinant human P4HB in a dose dependent manner. **(C)** The urinary KIM-1 levels in different groups of pristane induced mice ((control, n = 8; KLH, n = 8; KLH-HU1, n = 7). Data are shown as mean ± SD. Statistical analyses were performed with two-tailed unpair Student’s t test using GraphPad Prism 7 software. P >0.05 was considered nonsignificant.

We further investigated the effect of anti-HU1 on P4HB. Human P4HB was expressed (rhP4HB) and purified (**Figure S5A**). Interaction between anti-HU1 and rhP4HB was confirmed by ELISA (**Figure S5B**). Then, the effect of anti-HU1 on P4HB was determined by the insulin aggregation assay, a well-established assay for evaluating P4HB activity (**Figure 5A**). We detected the effect of anti-P4HB and Rb-anti-HU1 on P4HB activities, and both of antibodies were found to attenuate the insulin aggregation induced by P4HB (**Figures 5B**, **S5C**). As P4HB is located at the renal cell surface, we performed several assays to detect the effect of Rb-anti-HU1 on HEK 293T cells. However, there was no detectable effect of Rb-anti-HU1 on cell growth and apoptosis (**Figures S5D, E**).

It is reported that levels of urinary KIM-1 (kidney injury molecule-1) were higher in bacitracin (PDI inhibitor) treated rats compared to vehicle-treated control rats (26). We further detected the urinary levels of KIM-1 in pristane induced mice, and we found that the level of urinary KIM-1 in the mice of KLH-HU1 group was higher than those in KLH and control groups (**Figure 5C**).

## Discussion

The formation of aggregated and condensed structures by several proteins incorporated with DNA is documented to trigger the production of type I interferon and is involved the pathogenesis of autoimmune diseases (6, 17, 25). The antimicrobial peptide, LL37, binds DNA to form a coil-node structure and subsequently mediates pDC activation and drives autoimmunity in psoriasis (27–30). Moreover, Gallo PM et al. have demonstrated that amyloids from *Salmonella* form Curli fibers that bind tightly to eDNA in biofilms. Curli-DNA composites trigger the production of type I interferon in cDC and the production of autoantibodies in lupus-prone and wild-type mice (6). These reports suggest that proteins interacting with DNA may induce autoimmune disorders.

DNABII proteins are ubiquitously expressed in eubacteria that not only maintain the structure and function of chromatin, but also bind eDNA in biofilm as extracellular proteins (31). Until now, little is known about whether these proteins are involved in autoimmune disorders. Here, we have identified that DNABII proteins can trigger immune responses and induce the production of specific antibodies in most individuals. Although the level of antibodies against DNABII proteins in patients with autoimmune diseases is not different from that in healthy individuals, we identify a hexapeptide, HU1, derived from DNABII proteins that is one of the epitopes of DNABII proteins and is specifically recognized by sera from patients with SLE, but not from those with RA or pSS and healthy individuals. These results suggest that antibodies against DNABII proteins produced by different individuals recognize different epitopes. Only a portion of patients with SLE can produce antibodies against HU1 *via* an unresolved mechanism that needs to be addressed in the future.

Anti-HU1 can inhibit biofilm formation by *S. aureus*, suggesting that the production of anti-HU1 triggered by DNABII proteins is a protect mechanism against biofilm formation. Nevertheless, anti-HU1 cross-recognizes P4HB, a self-antigen located on the membrane of renal cells, and induces LN. This could be regard as an unwanted side effect induced by immune system.

P4HB located on the cell surface can catalyze the reduction of disulfide bonds in cell surface proteins (32). P4HB negatively regulates the shedding activities of cell surface ADAM17 that is a dis-integrin and metalloproteinase. Moreover, KIM-1 is reported to be shed from cell surface by ADAM17 (33). Our investigation showed that anti-HU1 can decrease the activities of P4HB. However, addition of anti-HU1 to renal cells (HEK293 cells or mouse primary kidney cells) has no observed effect on cell growth or apoptosis. We assumed that this autoantibody blocked the enzymic activities of P4HB and subsequently increased the level of soluble urinary KIM-1 by activation of ADAM17, which will be investigated in the future.

The kidney is affected in about 50% of patients with SLE (3). The development of LN is complex and unclear. Several immune events in the kidney are reported to contribute to the pathogenesis of LN including immune complex deposition, formation of neutrophil extracellular traps, and activation of the complement system (34–36). Our results show that the level of anti-HU1 is positively correlated with the occurrence of LN in patients with SLE and the presence of anti-HU1 aggravates the process of LN in the SLE murine model induced by pristane *in vivo*. All these results suggest that the production of anti-HU1 is a novel risk factor for the pathogenesis of LN.

Anti-dsDNA is a marker for the clinical diagnosis of SLE and is involved in the pathogenesis of this disease (37). The level of anti-HU1 is also positively related with the titer of anti-dsDNA in patients with SLE. However, the presence of anti-HU1 cannot alter the level of anti-dsDNA. Immunization with a complex of DNABII proteins and dsDNA does not induce production of anti-dsDNA (data not shown). Our observations indicate that interaction between DNABII proteins and DNA may not directly trigger the production of anti-dsDNA.

Our study shows that a novel autoantibody induced by ubiquitously expressed proteins in bacterial biofilm drives the development of LN by cross recognizing self-antigen. Our findings reveal a new mechanism involved in the pathogenesis of LN, and provide further evidence to the distinct relationship between the presence of pathogenic bacteria or commensal microbiota and the development of autoimmune diseases.

## Data Availability Statement

The original contributions presented in the study are included in the article/**Supplementary Material**. Further inquiries can be directed to the corresponding authors.

## Ethics Statement

The studies involving human participants were reviewed and approved by the ethics committee of the Peking University People’s Hospital. The patients/participants provided their written informed consent to participate in this study. The animal study was reviewed and approved by Animal Care and Use Committee of Beijing Institute of Pharmacology and Toxicology.

## Author Contributions

WF: acquired, analyzed, and interpreted data. YL: acquired and analyzed data. FL, CL, JL, JN, PH, DX and JH: provided administrative, technical, and material support. YM, JF and GL: supervised the study. RM: supervised the study and drafted the manuscript. GY: conceived and designed the study, obtained funding and drafted the manuscript. All authors contributed to the article and approved the submitted version.

## Funding

This work was supported by grants from National Natural Science Foundation of China (http://www.nsfc.gov.cn) [81871618] and Shandong Provincial Major Scientific and Technological Innovation Project (MSTIP)-2019JZZY011012. The funders had no role in study design, data collection and analysis, decision to publish, or preparation of the manuscript.

## Conflict of Interest

The authors declare that the research was conducted in the absence of any commercial or financial relationships that could be construed as a potential conflict of interest.
