# Corrigendum: A Novel Autoantibody Induced by Bacterial Biofilm Conserved Components Aggravates Lupus Nephritis

**DOI:** 10.3389/fimmu.2021.819846

**Published:** 2021-12-17

**Authors:** Wenyan Fu, Yu Liu, Fangjie Liu, Chenghua Liu, Jingjing Li, Jiali Niu, Peng Han, Dan Xu, Jiaojiao Hou, Yuanfang Ma, Jiannan Feng, Zhanguo Li, Rong Mu, Guang Yang

**Affiliations:** ^1^ Beijing Institute of Pharmacology and Toxicology, Beijing, China; ^2^ State Key Laboratory of Toxicology and Medical Countermeasures, Beijing, China; ^3^ Joint National Laboratory for Antibody Drug Engineering, Henan University, Kaifeng, China; ^4^ Department of Rheumatology and Immunology, People’s Hospital, Peking University, Beijing, China

**Keywords:** bacterial biofilm, DNABII, autoantibody, lupus nephritis, protein disulfide isomerase

In the original article, there was a mistake in **Figure 4C** as published. **Figure 4C** was incorrectly replaced with **Figure 4A** during the publishing process, which meant the two figures were the same. The corrected **Figure 4C** appears below.

**Figure 4:**
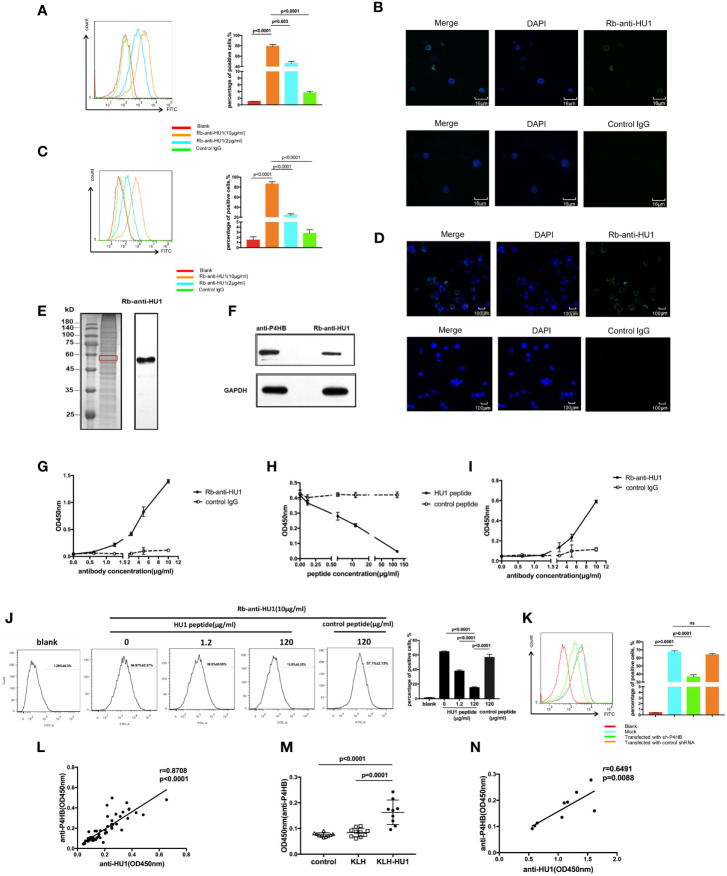
The protein disulfide isomerase, P4HB, as a target autoantigen. **(A)** Binding of Rb-anti-HU1 to mouse primary kidney cells was measured by flow cytometry. Live mouse primary kidney cells were stained with Rb-anti-HU1 (0 μg/ml-red, 2 μg/ml-blue, 10 μg/ml-orange), and then detected with DyLight™ 488 labeled donkey anti-rabbit IgG. Flow cytometry analysis was performed on FACSCalibur (Becton Dickinson). Data were processed using FlowJo software. Data are representative of three independent experiments and are shown as mean ± SD. **(B)** Confocal microscopy showed that Rb-anti-HU1 (10 μg/ml) recognizes a member antigen on mouse primary kidney cells. **(C)** Binding of Rb-anti-HU1 to HEK293T cells was measured by flow cytometry. HEK293T cells were stained with Rb-anti-HU1 (0 μg/ml-red, 2 μg/ml-blue, 10 μg/ml-orange), and then detected with DyLight™ 488 labeled donkey anti-rabbit IgG. Flow cytometry analysis was performed on. FACSCalibur (Becton Dickinson) and data were processed using FlowJo software. Data are representative of three independent experiments and are shown as mean ± SD. **(D)** Confocal microscopy showed that Rb-anti-HU1 (10 μg/ml) recognizes a member antigen on HEK293T cells. **(E)** HEK293T cells total proteins were extracted and detected by western blot using Rb-anti-HU1 antibody. The band labeled in the red square indicates the specific band recognized by Rb-anti-HU1. Data represent one of three independent experiments. **(F)** Bands recognized by Rb-anti-HU1 and Mo-anti-P4HB were detected from total proteins of HEK293T cells by western blot. The interaction between Rb-anti-HU1 and human P4HB **(G)** and mouse P4HB **(I)** was measured by ELISA. Data are shown as the mean ± SD. **(H)** Specific binding of Rb-anti-HU1 to human P4HB was blocked by HU1 peptide in a concentration-dependent manner. Data are shown as mean ± SD. **(J)** Specific binding of Rb-anti-HU1 to native P4HB on mouse primary kidney cell surface was blocked by HU1 peptide in a concentration-dependent manner. Data are representative of three independent experiments and are shown as mean ± SD. **(K)** P4HB expression was knocked down by a specific small hairpin RNA(sh-P4HB). Flow cytometry was then performed to detected the specific binding of Rb-anti-HU1 (5 μg/ml) to P4HB on the surface of HEK293T cells by flow cytometry. Data are shown as mean ± SD. **(L)** Correlation between the anti-HU1 antibody and anti-P4HB antibody in sera from patients with SLE. Each point represents a measurement for an individual patient (n = 62). **(M)** Detection of anti-P4HB titer in the sera of mice immunized with KLH-HU1 and control groups by ELISA at week 23 post-pristane induction (control, n = 10; KLH, n = 10; KLH-HU1, n = 9). **(N)** Correlation between anti-HU1 antibody and anti-P4HB antibody in sera from mice immunized with KLH-HU1 (n = 9). Data are presented as means ± SD. The differences between two groups were statistically analyzed with two-tailed unpair Student’s t test using GraphPad Prism 7 software. The correlation between two indicators were statistically analyzed with correlation analysis using GraphPad Prism 7 software. P >0.05 was considered nonsignificant.

The authors apologize for this error and state that this does not change the scientific conclusions of the article in any way. The original article has been updated.

## Publisher’s Note

All claims expressed in this article are solely those of the authors and do not necessarily represent those of their affiliated organizations, or those of the publisher, the editors and the reviewers. Any product that may be evaluated in this article, or claim that may be made by its manufacturer, is not guaranteed or endorsed by the publisher.

