# Supplementary material for: A Novel Autoantibody Induced by Bacterial Biofilm Conserved Components Aggravates Lupus Nephritis
Source: Front Immunol. 2021 Mar 25;12:656090. doi: 10.3389/fimmu.2021.656090 (PMC8027312; doi:10.3389/fimmu.2021.656090)
Supplement: Supplementary file 1 [file DataSheet_1.pdf]

Figure S1

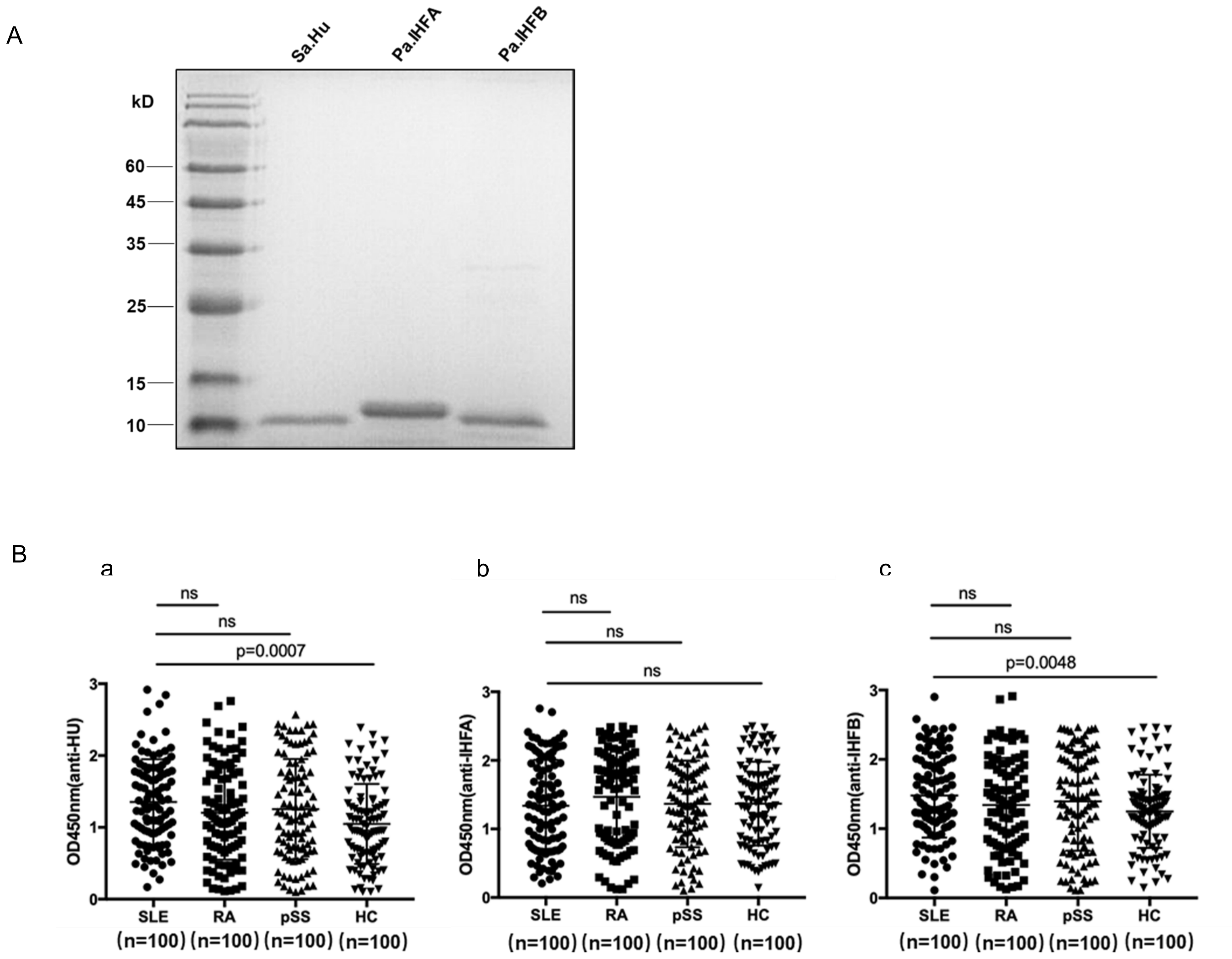

Figure S1, related to Figure 1.

(A)three recombinant DNABII proteins (Sa.HU, Pa.IHFA, and IHFB) were analyzed by sodium dodecyl sulfate-polyacrylamide gel electrophoresis (SDS-PAGE). These proteins have a molecular weight of approximately 10 kD. (B)Levels of antibodies against Sa.HU(a), Pa.IHFA(b), and Pa.IHFB(c) in sera from patients with SLE (n = 100), RA (n = 100), pSS (n = 100), and from HC (n = 100). Each point represents a measurement for an individual. Statistical analyses were performed with two-tailed unpair Student’s t test using GraphPad Prism 7 software. P>0.05 was considered nonsignificant. All data are presented as means $\pm$ SD.

Figure S2

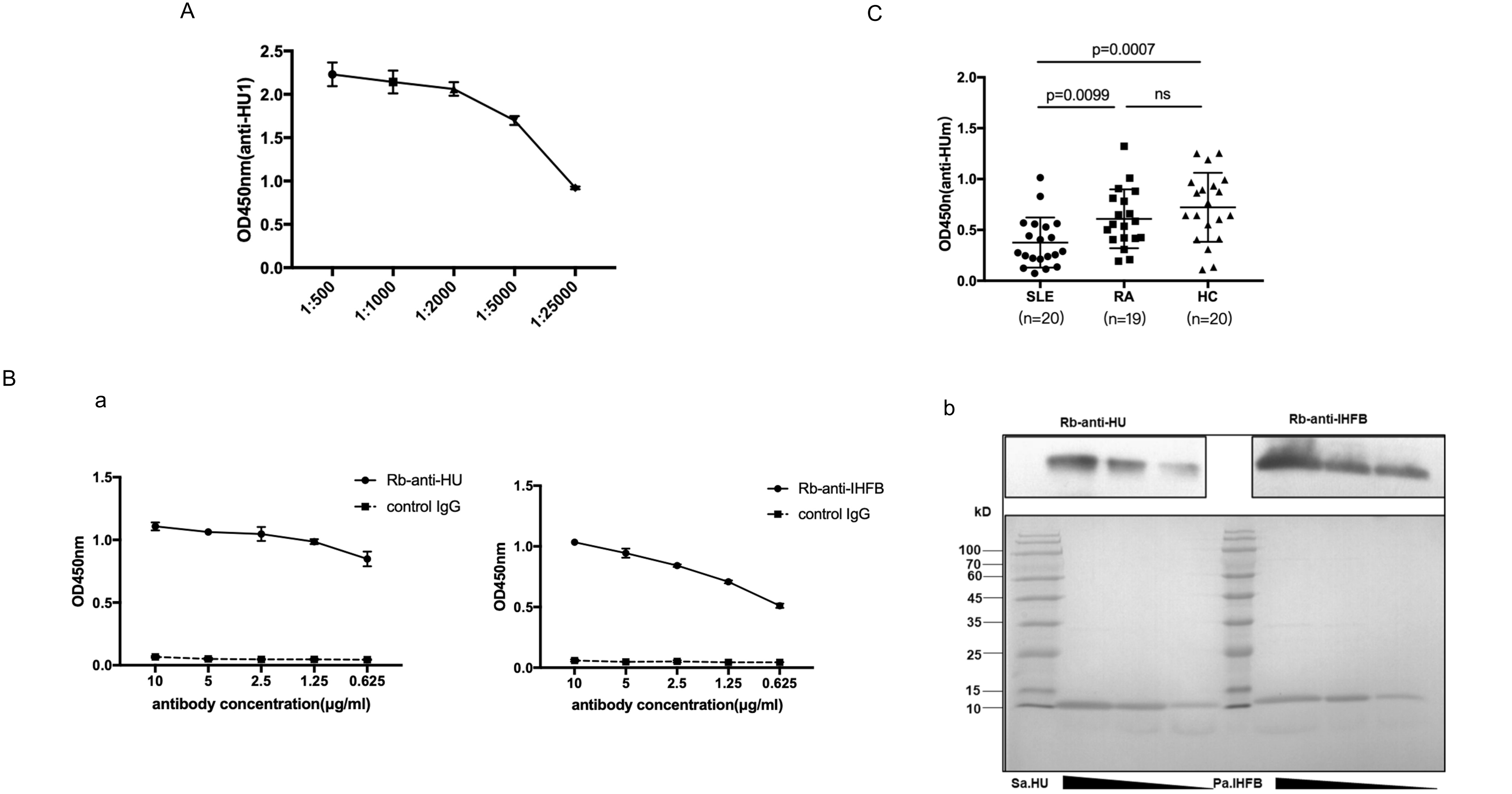

Figure S2, related to Figure 2.

(A)ELISA for the detection of anti-HU1 antibody levels in rabbit antiserum obtained from a KLH-HU1 immunized rabbit at different dilution ratios (from 1:500 to 1:25000). Data are representative of three independent experiments and are shown as mean ± SD. (B)ELISA (a) and western blot (b, upper) showing the specific recognition of Rb-anti-HU and Rb-anti-IHFB with Sa.HU and Pa.IHFB individually. Sa.HU and Pa.IHFB (10 μg, 5 μg,1 μg) were analyzed by SDS-PAGE(b, lower).(C) Levels of antibodies against Sa.Hum in sera from patients with SLE (n=20), RA (n=20) and pSS (n=20). Each point represents a measurement for an individual. Statistical analyses were performed with two-tailed unpair Student's t test using GraphPad Prism 7 software. P>0.05 was considered nonsignificant. All data are presented as means ± SD.

Figure S3

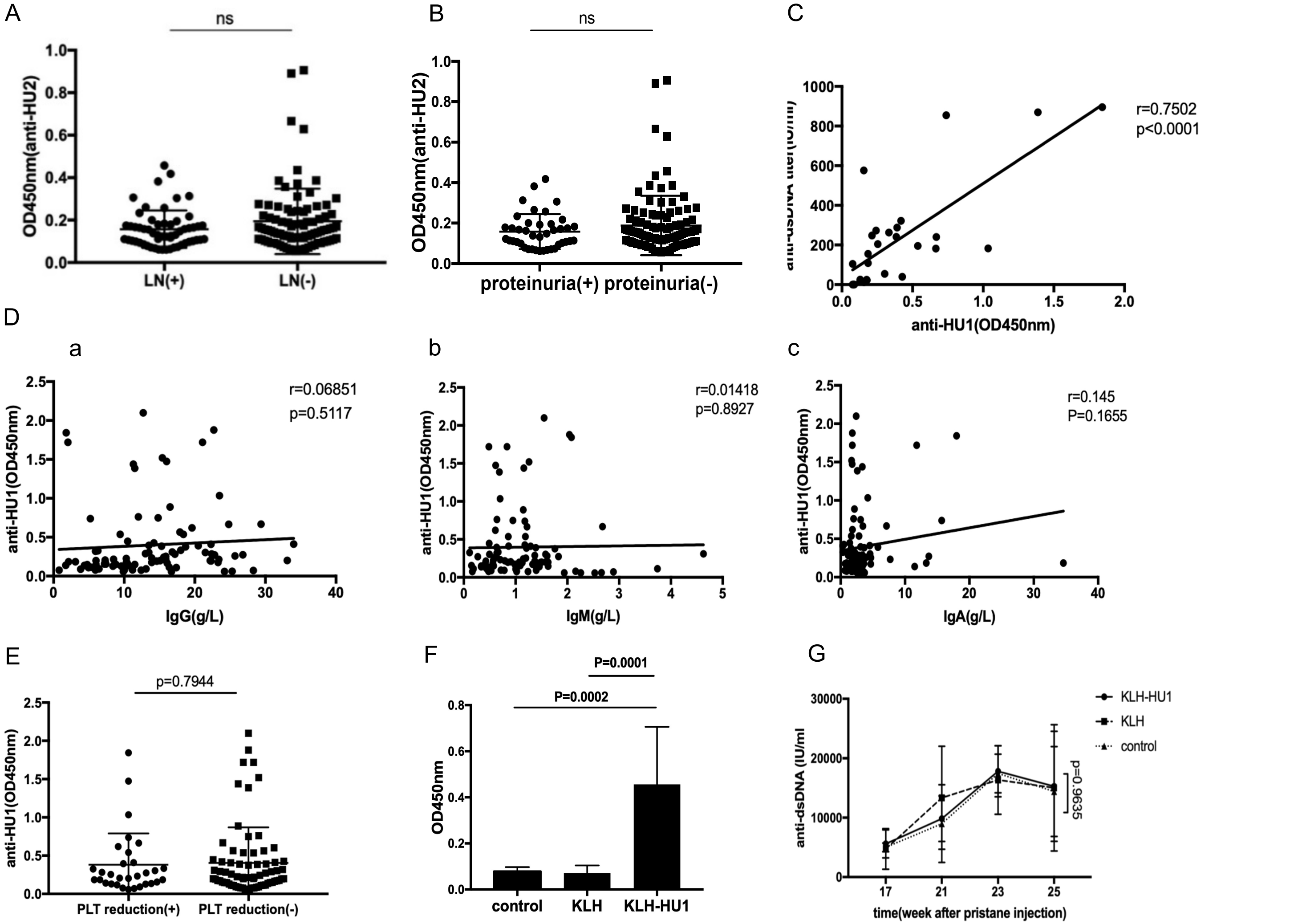

Figure S3, related to Figure 3.

(A) Correlation between anti-HU2 antibody in SLE patients with LN and patients with SLE alone.(B) Correlation between anti-HU2 antibody in SLE patients with proteinuria and without proteinuria. Statistical analyses were performed with two-tailed unpair Student's t test using GraphPad Prism 7 software. P>0.05 was considered nonsignificant**.** **(**C)Correlation between anti-HU1 antibody and anti-dsDNA antibody in sera from patients with SLE according to their clinical features.(D)Correlation between anti-HU1 antibody and concentration of IgG (a), IgM (b), and IgA (c) in sera from patients with SLE. Statistical analyses were performed with correlation analysis using GraphPad Prism 7 software. P>0.05 was considered nonsignificant. (E) Correlation between anti-HU1 antibody and reduction of platelets (PLT) in patients with SLE. Statistical analyses were performed with two-tailed unpair Student's t test using GraphPad Prism 7 software. P>0.05 was considered nonsignificant. (F) Level of anti-HU1 in the sera(1:800) of mice immunized with KLH-HU1 and control groups by ELISA after 5 times of immunization(n=10,control; n=10,KLH; n=10,KLH-HU1). (G)Detection of anti-dsDNA antibody in sera of KLH-HU1 immunized mice and control groups mice at the indicated time. Statistical analyses were performed with a two-way ANOVA test using GraphPad Prism 7 software. P>0.05 was considered nonsignificant. Data are shown as mean ± SD.

Figure S4

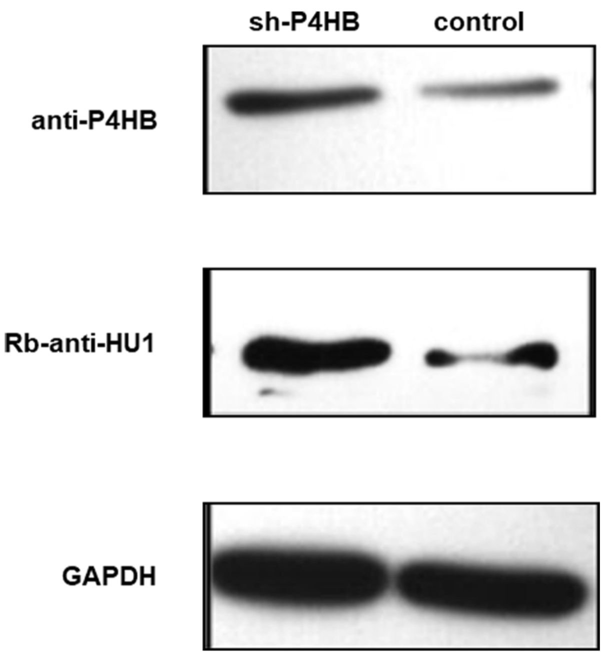

Figure S4, related to Figure 4. The expression of P4HB in HEK293T cells was inhibited by a specific small hairpin RNA targeting P4HB (sh-P4HB) by western blot.

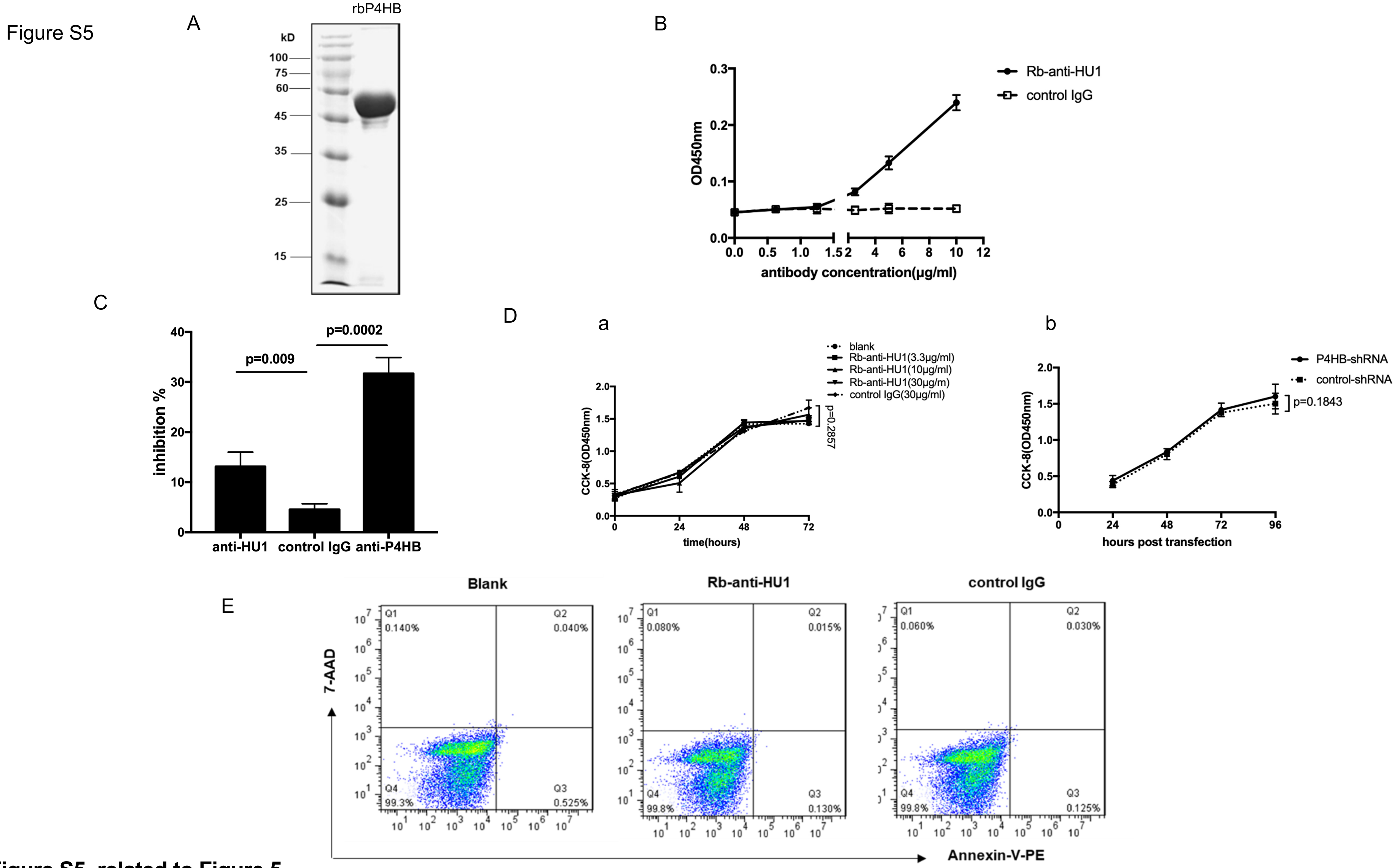

Figure S5, related to Figure 5.

(A)Purified recombinant human P4HB (rhP4HB) was analyzed by SDS-PAGE. (B)Interaction between Rb-anti-HU1 and rhP4HB was detected by ELISA. Data are representative of three independent experiments and are shown as mean ± SD. (C)Rb-anti-P4HB(0.15μg/ml) inhibited the activity of P4HB more effectively compared with the same concentration of anti-HU1. Statistical analyses were performed with two-tailed unpair Student's t test using GraphPad Prism 7 software. P>0.05 was considered nonsignificant(D)CCK8 assay was used to evaluate the proliferation of HEK293T cells after Rb-anti-HU1 treatment (a) and after transfection with the sh-P4HB or control shRNA (b). Statistical analyses were performed with a two-way ANOVA test using GraphPad Prism 7 software. P>0.05 was considered nonsignificant.(E)After incubation with Rb-anti-HU1 (0, 30 μg/ml) or control IgG(30 μg/ml) for 24 h, HEK293T cells were collected and stained with both Annexin-V-PE and 7-AAD for apoptosis analysis.

Table S1. Demographic Characteristics of the RA, pSS Patients and Controls

Diagnose	No. of patients	Mean Age	Gender
		(Range)	Male/Female
		yr	No.
RA	136	52(17-79)	30/106
pSS	133	56(18-79)	5/128
healthy donor	150	45(22-60)	25/125

Table S2. Demographic Characteristics of the SLE Patients

Diagnose	No. of patients	Lupus nephritis		Mean Age	Gender
				(Range)	Male/Female
		positive	negative	yr	No.
SLE	103	61	42	38(13-75)	8/95

Table S3. Top 20 candidates identified by mass spectrometry from HEK293T cell lysis

Rank	Protein	Mass(Da)	score	Expressed on Cell membrane
1	PERM_HUMAN	84784	4600	no
2	PLSL_HUMAN	70814	2173	yes
3	G6PI_HUMAN	63335	1656	no
4	TRFL_HUMAN	80014	1625	no
5	KPYM_HUMAN	58470	1609	no
6	CATA_HUMAN	59947	1482	no
7	K1C10_HUMAN	59020	1289	no
8	K2C1_HUMAN	66170	1273	yes
9	MYH9_HUMAN	227646	1187	no
10	K22E_HUMAN	65678	1035	yes
11	FIBB_HUMAN	56577	919	no
12*	***PDIA1_HUMAN***	***57480***	***912***	***yes***
13	COR1A_HUMAN	51678	818	no
14	CAP1_HUMAN	52325	732	yes
15	PDIA3_HUMAN	57146	690	no
16	ILEU_HUMAN	42829	566	no
17	HS71A_HUMAN	70294	561	no
18	PERE_HUMAN	81985	544	no
19	K1C9_HUMAN	62255	474	no
20	TKT_HUMAN	68519	470	no

*PDIA1(P4HB) is the only transmembrane protein with ~58 kDa molecular weight

Table S4. primers used in the study for PCR

Protein	Forward Primer(5'-3')	Reverse Primer(5'-3')
SA.HU	GCTAGCATGAACAAAACAGATTTAAT	CTCGAGTTATTTTACAGCATCTTTTA
PA.IHFA	GCTAGATGACCAAGTCGGAGTTG	CTCGAGTTATGACTTGGTTCCAGCA
PA.IHFB	GCTAGCATGACCAAGTCGGAGTTGATCGAA	CTCGAGTCACTCCGGCTC
SA.HUM	GCTAGCATGAACAAAACAGATTTAAT	CTCGAGTTATTTTACAGCATCTTTTAATGCTTTACCAGCTTTGAATGCTGGAACTTTACTTGCTGGGATATCAATTTCTTTACCTGCTGCTGCTGCTGCTGCTTTACGTGCAGCACGTT
PA.IHFAM	GCTAGATGACCAAGTCGGAGTTG	CTCGAGCTCCGGCTCGTTGACCCGATCCCGCAACTCCTTGCCCGGCTTGAAGTGCGGCACGAACTTGCCGTCGAGGCGTACCGACTCCCCGTCTGCTGCTGCTGCTGCGACGCGCGGGG
PA.IHFBM	GCTAGCATGACCAAGTCGGAGTTGATCGAA	CTCGAGTCACTCCGGCTCGTTGACCCGATCCCGCAACTCCTTGCCCGGCTTGAAGTGCGGCACGAACTTGCCGTCGAGGCGTACCGACTCCCCTGCTGCTGCTGCTGCTGCGACGCGCGG
